# Technologies for High-Throughput Identification of Antibiotic Mechanism of Action

**DOI:** 10.3390/antibiotics10050565

**Published:** 2021-05-12

**Authors:** Bernardo Ribeiro da Cunha, Paulo Zoio, Luís P. Fonseca, Cecília R. C. Calado

**Affiliations:** 1 Institute for Bioengineering and Biosciences (iBB), Instituto Superior Técnico (IST), Universidade de Lisboa (UL), Av. Rovisco Pais, 1049-001 Lisboa, Portugal; bernardo.cunha@tecnico.ulisboa.pt (B.R.d.C.); pjzoio@gmail.com (P.Z.); luis.fonseca@tecnico.ulisboa.pt (L.P.F.); 2 CIMOSM—Centro de Investigação em Modelação e Optimização de Sistemas Multifuncionais, ISEL—Instituto Superior de Engenharia de Lisboa, Instituto Politécnico de Lisboa, R. Conselheiro Emídio Navarro 1, 1959-007 Lisboa, Portugal

**Keywords:** antibiotic discovery, bacterial cytological profiling, chemical genetics, high-throughput screening, Mechanism-of-Action (MOA), metabolomics, phenotypic screening, proteomics, transcriptomics, vibrational spectroscopy

## Abstract

There are two main strategies for antibiotic discovery: target-based and phenotypic screening. The latter has been much more successful in delivering first-in-class antibiotics, despite the major bottleneck of delayed Mechanism-of-Action (MOA) identification. Although finding new antimicrobial compounds is a very challenging task, identifying their MOA has proven equally challenging. MOA identification is important because it is a great facilitator of lead optimization and improves the chances of commercialization. Moreover, the ability to rapidly detect MOA could enable a shift from an activity-based discovery paradigm towards a mechanism-based approach. This would allow to probe the grey chemical matter, an underexplored source of structural novelty. In this study we review techniques with throughput suitable to screen large libraries and sufficient sensitivity to distinguish MOA. In particular, the techniques used in chemical genetics (e.g., based on overexpression and knockout/knockdown collections), promoter-reporter libraries, transcriptomics (e.g., using microarrays and RNA sequencing), proteomics (e.g., either gel-based or gel-free techniques), metabolomics (e.g., resourcing to nuclear magnetic resonance or mass spectrometry techniques), bacterial cytological profiling, and vibrational spectroscopy (e.g., Fourier-transform infrared or Raman scattering spectroscopy) were discussed. Ultimately, new and reinvigorated phenotypic assays bring renewed hope in the discovery of a new generation of antibiotics.

## 1. Introduction

Antibiotics have significantly improved many aspects of society. From their application in medicine resulted an increase of life expectancy and well-being, without which even the simplest of medical interventions would pose life-threatening risks [1]. However, antibiotic discovery has stagnated at alarmingly low rates since its golden age, when most classes in use today were discovered. Infectious disease in general, and multidrug resistant pathogens in particular, are increasingly a worldwide concern, and many calls for action have been issued, especially to reiterate the desperate need for new drugs [2].

There are two main antibiotic discovery strategies, target-based and phenotypic screening. While the target-centric approach begins with a pre-defined target whose inhibition should result in the desired therapeutic effect, phenotypic screening starts with a cell-based assay that monitors a phenotype, e.g., growth inhibition [3]. While phenotypic screening has a higher likelihood of identifying candidate drugs, along those that target poorly understood biological pathways, their molecular targets are not identified in the process and require subsequent efforts [4]. This results in higher rates of rediscovery, which is a key challenge in natural product antibiotic discovery [5] and an inability to detect low potency candidates, which can be later modified for enhanced therapeutic effect [6].

**Figure 1:**
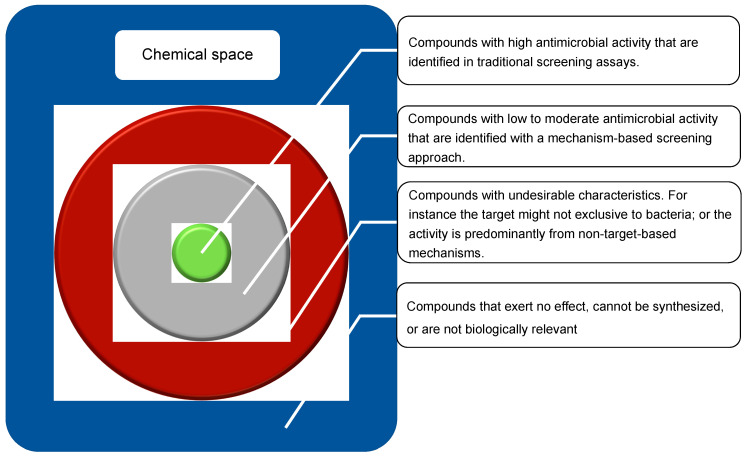
Representation of the chemical space (blue), including relevant subsets, such as those have undesirable characteristics for antibiotics (red), those that induce some level of phenotypic modulation but are not detectable with traditional screening (grey), and easily identified compounds with high-antimicrobial activity (green).

Although finding new antimicrobial compounds is very challenging, identifying their mechanism of action (MOA) has proven equally defiant [7]. For instance, the MOA of penicillin is still subject to debate, with recent studies suggesting a more complex mechanism than inhibition of cell wall synthesis [8]. As such, technologies to determine the MOA are a major bottleneck of antibiotic discovery. Currently, screening hundreds of thousands of compounds is a reasonable throughput of a drug discovery program, in part due to the ease in synthetizing bioactive compounds, and in part given the increasing availability of natural product libraries [9]. This is particularly important because knowledge on the exact molecular target, and the pathways it is involved in, facilitates lead optimization by rapidly excluding derivatives with increased activity due to off-target effects [10], thereby guiding medicinal chemistry programs towards improved chances of commercialization [11]. Moreover, the ability to rapidly detect MOA could enable a shift from the activity-based discovery paradigm towards a mechanism-based approach. This would expand the screenable chemical space by enabling the detection of compounds with low antimicrobial activity, the designated grey chemical matter, i.e., compounds that induce phenotypic modulation without sufficient potency to be detected in traditional screening assay (Figure 1). The grey chemical space is an underexplored source of structural novelty that, after structural optimization, could yield much needed new antibiotics [12].

Conventional MOA studies are based on macromolecular synthesis assays, which measure radioactively labeled molecular precursors to ascertain the inhibition of DNA, RNA, protein, lipid, or peptidoglycan synthesis. This implies that compounds which act on different steps of the same pathway cannot be distinguished, thereby missing out on potentially novel MOA. Additionally, all pathways are apparently impacted by compounds which kill bacteria very rapidly, such as disinfectants, even though they often affect specific pathways. To make matters worse, these assays are typically slow, laborious, low resolution, low accuracy, and low throughput [13]. Alternatively, biochemical approaches, like affinity chromatography, can identify the exact biomolecule to which a compound binds [14,15], but only in the case of a high-affinity small molecule and a fairly abundant protein receptor [16]. Moreover, these require large quantities of test compound, which are not always available in the early stages of the discovery process where screening is paramount. As such, novel methods capable of probing this complex phenomena are urgently needed to ease the process of antibiotic discovery [17]. In this study, several applications of system-wide profiling techniques for MOA identification were reviewed. Particular attention was given to techniques with sufficient throughput to be employed in screening campaigns of large libraries, whose advantages and limitations were described in light of several examples.

## 2. Chemical Genetics

**Figure 2:**
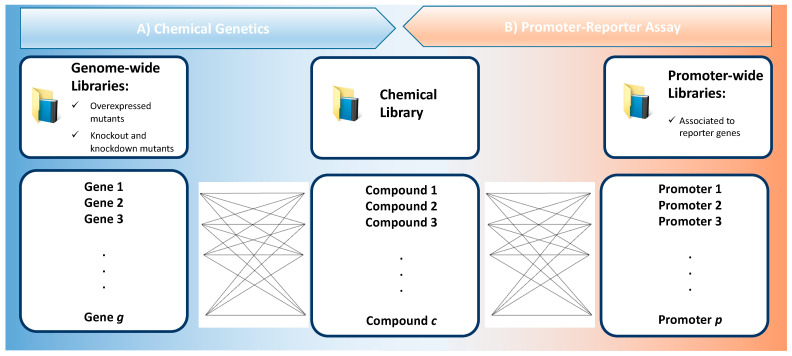
High-throughput screening assays based on genome-wide (**A**) or promoter-wide (**B**) bacterial mutant libraries that are tested against a chemical library. Ideally, the total number of genes (g) or promoters (p) considered in a screening campaign are minimized to reduce the workload, although this often yields less information. On the other hand, the diversity of all the compounds that constitute the chemical library (c) should be increased, as this improves the likelihood of discovering novelty.

At its core, chemical genetics evaluates a library of chemical compounds against a genome wide library, i.e., it maps the effect of a wide set of exogenous ligands across a wide set of genetic variants of cells models [18] (Figure 2A). The genomic library can be based on mutants with a gain of function or with loss of function. When the exogenous ligand is an antibiotic candidate, the effect across a mutant library enlightens its MOA. Interestingly, querying the MOA of antibiotics with chemical genetics contributed to our comprehension of many microbial processes, such as the synthesis of nucleic acids, proteins, and the cell wall [19].

### 2.1. Overexpression Libraries

Identifying MOA with overexpression libraries involves screening mutants that, when exposed to a compound that targets the product of an overexpressed gene, display a resistant profile. In other words, if the target is overexpressed, a larger antibiotic dose is required compared with a wild-type strain. Additionally, because challenging these resistant collections with antibiotics generates unique mechanistic fingerprints of the multilevel interactions induced, these assays reveal some, if not all, the participants in the network targeted by the antibiotic. The first published application of resistant libraries to ascertain antibiotics MOA in a high-throughput phenotypic screening assay was attempted by Li et al. [20], who screened a 8640 small molecule commercial collection for growth inhibitors of a 20,000 random mutant library of *Escherichia coli* MC1061. The plasmids in these clones were sequenced and two genes were identified, *folA* and *acrB*, which translate to dihydrofolate reductase and the (multidrug) acridine efflux pump. While the first was the target of two similar compounds, the latter surprisingly could efflux the remaining compounds. Given the nature of multicopy suppression assays, one hurdle is filtering out genes that code some drug-resistance mechanism from those that code the molecular target of compounds. In addition, despite it being a high-throughput assay, the library size is a clear disadvantage for largescale campaigns.

Building on the previous study, the ASKA collection was constructed. ASKA is an ordered high-expression *E. coli* library containing (nearly) all ORFs from the K12 W3110 strain in pCA24N high copy number plasmids [21]. As such, both essential and non-essential genes can be queried in regard to overexpression. Using ASKA, Pathania et al. [22] screened ~50,000 small molecules at a range of concentrations, which allowed a stringency-type analysis, whereby suppression of growth inhibition by a given mutation was evaluated along drug dose, therefore proving a more precise identification of the main molecular target at high drug doses, but also revealing other secondary targets at lower doses. At high-stringency (16xMIC), the targets of fosfomycin, fosmidomycin, trimethoprim, sulfamethoxazole, and D-cycloserine were clearly identified, but not of spectinomycin, whose target only became apparent at lower stringencies (8xMIC). More importantly, high-stringency analysis identified MAC13243, a novel compound whose target is the periplasmatic protein LolA, responsible for lipoprotein transport across the periplasmic region. MAC13243 represented a novel promising antibacterial, whose target and MOA belonged to a (then) novel class and thus warranted further investigation.

Later studies into the degradation of MAC13243 revealed that the breakdown product S-(4-chlorobenzyl)isothiourea was responsible for its antibacterial activity, and this compound is in fact a structural analogue of S-(3,4-dichlorobenzyl)isothiourea, whose ability to disrupt the actin-like cell shape-determining MreB protein had already been reported [23]. Because MAC13243 breaks down in aqueous medium, its use as a lead molecule was questioned. However, its alternative use as a permeabilization agent, to potentiate large-scaffold antibiotics, has been suggested, although further structural optimization is likely required [24]. Despite the limitations of MAC13243 as a therapeutic agent, its target, LolA, is part of a five-protein system (LolABCDE) that is an attractive target of Gram negative pathogens. Since the outer membrane of Gram negative bacteria is a permeability barrier, it confers greater structural integrity, and participates in a panoply of other roles, including the translocation of proteins and nutrients, adhesion and signal transduction. It is not only essential for survival, but also more easily accessible to drugs in comparison with cytoplasmatic targets [25].

### 2.2. Knockout and Knockdown Collections

In contrast with overexpression libraries, the Keio collection is single-gene knockout library of *E. coli* K12 BW25113, where the kanamycin resistance cassette takes the place of the deleted gene [26]. Being a knockout library, only non-essential genes can be probed. Although not as useful for MOA identification as overexpression libraries, the Keio collection highlighted the potential of combinatorial therapies [27]; contributed towards the characterization of gene essentiality and chromosomal organization [28]; revealed the complex interplay of metabolic pathways elicited during nutrient stress, which elucidated gene function and unwrapped new antibiotic targets [29]; and illuminated mechanisms of resistance, including determinants of drug permeability, efflux, degradation as well as stress responses [30]. Stokes et al. [31] used the Keio collection to comprehend how *E. coli* became susceptible to vancomycin, a narrow-spectrum antibiotic active against Gram positive bacteria, under cold stress. While this hydrophilic antibiotic is unable to pass the outer membrane of Gram negative bacteria, transient ‘cracks’ in the outer membrane caused by low temperatures allow its diffusion into the periplasm, allowing it to reach its target.

While controlling essential gene dosage by knockout mutations is technically accessible in diploid eukaryotic organisms, for instance via genome-wide haploinsufficiency profiling [32], this is more challenging in prokaryotes, and early studies were limited to a low number of genes [33]. Probing essential genes requires a conditional knockdown, e.g., the use of mutants that only display a mutant phenotype in a given restrictive condition [34]. The first hypersensitized microbial collection that allowed the modulation of essential genes used xylose-inducible antisense RNA expression in *Staphylococcus aureus*. Here, 245 target-depleted strains could be tuned to control essential gene expression to obtain moderate growth suppression (~20%) through to the knockout phenotype. As such, comparison of a hypersensitized phenotype with that obtained after exposure to a compound reveals its target [35]. Using this collection, Phillips et al. [5] conducted a natural product screening program that revealed kibdelomycin, a novel type II topoisomerase inhibitor. Given its broad spectrum of activity, especially against Gram positive bacteria, along lack of cross-resistance, there was great expectation for kibdelomycin, which so far has not materialized into new drugs reaching the market.

Gene downregulation with antisense RNA is not without limitations. In cases involving polycistronic mRNA, the entire strand may be degraded when the antisense RNA binds, resulting in the suppression of more genes than desired. In cases where there are common motifs, undesirable gene suppression may also occur [36]. A more specific and efficient methodology for gene knockdown relies on Clustered Regularly Interspaced Short Palindromic Repeats/dCas9 (CRISPR) transcriptional regulation. Peters et al. [37] employed CRISPR to develop a knockdown library of *Bacillus subtilis* also modulated by xylose, but in this case the plasmids were integrated in the microbial chromosome. With this technique, the MOA of MAC0170636 was identified, namely the inhibition of undecaprenyl pyrophosphate synthetase, which is essential for cell wall synthesis. Interestingly, some gene annotations are being revised with CRISPR. Liu et al. [38] characterized the function of previously ‘hypothetical’ genes of *Streptococcus pneumoniae*. However, only 73% of genes deemed essential with transposon sequencing were considered essential with CRIPSR knockdown. While this could be due to inadequate annotation using transposon sequencing, it could also be due to different growth conditions, or to insufficient suppression with CRISPR. Regardless, given CRISPR systems are found across a range of bacteria and can be easily transferred to other microorganisms, constructing similar downregulated libraries could be a promising step forward towards novel antibiotics and better therapeutics.

## 3. Promoter-Reporter Libraries

Promoter-reporter libraries are used to build a map of how antibiotics affect the activation of a high number of promoters. For that, a target gene is used to screen over a high dimension of promoters (Figure 2B). Comparison with baseline transcriptional levels reveals the differential effects induced by antibiotics on that promoter, and doing so across multiple strains in a library, maps out the global response that can be used as a MOA profile [9], as well as to query cellular pathways and unravel off-target effects [39]. Because a fluorescent or luminescent signal is produced with the transcription of the promotor-reporter gene, this technique offers adequate temporal resolution to study the effect of antibiotic exposure through a time course [19]. In comparison with fluorescence-based assays, luminescence is typically preferred for screening purposes given bacteria’s considerable autofluorescence, which adds background noise. As such, luciferase reporters as luxCDABE are preferred given their low background noise, capacity to screen small colonies on solid media, with high reproducibility, without the need for substrate addition for continuous signal. Nonetheless, luciferase synthesis requires ATP and is affected by the redox potential, as such some false positives occasionally occur.

Zaslaver et al. [40] developed an *E. coli* K12 MG1655 library where the Green Fluorescent Protein (GFP) gene was fused to ~1820 different promoter regions, which is over 75% of known promoters, in low copy-number plasmids pUA66. This allowed highly accurate near- genome-wide measurements of promoter activity. Nonetheless, some transcriptional activity outside the individual promoter was occasionally detected. This limitation of promoter-reporter assays is independent of the reporter gene chosen. Even so, this library revealed the mechanism ruling suppression antagonism of DNA and protein synthesis inhibitors, where DNA stress responses result in nonoptimal regulation of ribosomal genes, distorting the DNA-to-protein ratio, and suppressing protein synthesis inhibition [41].

More recently, promoter-reporter collections have been used to characterize stress responses elicited by bioactive compounds. To that end, Elad et al. [39] constructed a panel of 15 *E. coli* strains with the luxCDABE plasmid, to which promoters elicited during particular stresses were inserted. These stresses include DNA damage, protein misfolding, inhibition of fatty acid synthesis, increase of reactive oxygen species, and the presence of metals. Of the 420 FDA-approved drugs tested, 89 elicited a response, even though some were not directed at prokaryotes. Interestingly, these responses were clustered in accordance to drug class, and often predicted their toxicity. In line with this, the collection produced by Zaslaver et al. was challenged with nine antibiotics, which revealed the cellular pathway affected, off-target effects, and to some extent the MOA.

## 4. Transcriptomics

Transcripts were initially characterized with northern blotting or Polymerase Chain Reaction (PCR), but these assays were mostly limited to a few transcripts. The development of microarrays in the 1990s enabled the quantitative detection of most known transcripts of a strain in a defined condition using a single assay [42]. In practice, microarrays reveal the hybridization between oligonucleotides of the reference strain and its labeled complementary DNA obtained from an experimental strain, e.g., one that has been exposed to an antibiotic. As such, microarrays are limited to strains whose genome is known and available when developing the microarray. Alternative hybridization-based techniques include high-density bead arrays, electronic microarrays, or suspension bead arrays, which have been revised elsewhere [43].

### 4.1. Hybridization Assays

Boshoff et al. [44] used whole-genome microarrays to measure the effect of various drugs, along with growth-inhibitory conditions, on *Mycobacterium tuberculosis*. Transcripts with at least a three-fold change were analyzed and clustered according to their known regulatory network. These were coherent with the known MOA of inhibitors of cell-wall synthesis, protein synthesis, transcription, and DNA gyrase. Moreover, these clusters also suggested the MOA of uncharacterized compounds, including a natural product extract in either its crude or purified form, whose MOA was later identified by reporter strains and biochemical assays. Similarly, Liang et al. [45] explored the transcriptional response of *M. tuberculosis* to linezolid, which binds to the 50S ribosomal subunit and inhibits protein synthesis. In total, 729 genes were differentially expressed, including genes involved in protein synthesis, sulfite metabolism, cell-wall synthesis, among others. Surprisingly, genes closely related to linezolids’ target were down-regulated. This reveals the complexity of transcriptional responses to antibiotics, as well as the challenge of pinpointing the biomolecular target of the drug. For example, Bonn et al. [46] identified a similar number of genes with differential expression; however, the genes closely related to linezolids’ target were found to be up-regulated. Although hybridization assays provide reasonable throughput at a relatively low cost, issues regarding reliability and reproducibility are well known. For instance, only high copy transcripts are easily detected, outputs often have high background noise due to cross-hybridization, signal saturation is common, and several issues arise given that probes are based on predicted open reading frames of sequenced genomes [47].

### 4.2. The Uprising of Next-Generation Sequencing

Many of the disadvantages of microarrays can be overcome with sequencing strategies. Initially reliant on the Sanger method, and later variants thereof, sequencing techniques were limited by high cost, inability to map short reads, and incomplete transcript sequencing [48]. Next-generation sequencing (NGS) allowed the analysis of larger sequence numbers, with higher reproducibility, from strains whose genome need not be sequenced. Moreover, RNA-seq has little to no background noise and virtually no saturation [48]. Recently the cost of RNA-seq has been steadily decreasing into reasonable levels, which explains its increasingly widespread application [49]. Many transcriptomic studies in the RNA-seq era focus on the particular transcriptional response of a single compound [50]. By design, these studies were descriptive, rather than predictive, of MOA. Nevertheless, these have consolidated the correlation between MOA and transcriptional response, thereby established transcriptomics as an essential tool in antibiotic discovery. Because many of the said studies have been revised elsewhere [51,52], the focus of this section shifts to RNA-seq studies that compare different MOA with a predictive approach.

One of the advantages of RNA-seq is the ability to probe non-coding RNA. For instance, Howden et al. [53] explored the role of both the mRNA and sRNA of a multidrug-resistant *S. aureus* after exposure to four last-resort antibiotics. Interestingly, mRNA profiles more closely reflected growth conditions, and the strain analyzed, than antibiotic exposure, except for the linezolid-induced transcriptional response, where a clear profile emerged. On the other hand, 39 differently expressed sRNAs confirmed with northern blotting provided better MOA profiles. Moreover, it is interesting to note that many antisense sRNAs associated with protein synthesis genes were down-regulated independently of the drug tested. Similarly, Molina-Santiago et al. [54] explored the transcriptional response induced by eight antibiotics on *Pseudomonas putida*, which was chosen as a model organism given its resistance to high concentrations of various antibiotics. In total, 5756 mRNAs, 58 tRNAs, and 154 sRNAs were identified. Two-fold changes in mRNA levels resulted in two clusters, one with kanamycin, ampicillin, and chloramphenicol, all of which had similar profiles to the control samples. The second cluster presented more distinct transcriptional responses regarding the control. Using sRNA profiles, only ampicillin and chloramphenicol clustered together with the controls, suggesting sRNA profiles are better suited for MOA classification. Sequencing-based transcriptomics also allows to study the pathogen within the host, which reveals microbial gene expression during infection. For instance, the adaptation of *Mycobacterium* inside macrophages as disease progresses, but also the efficacy of treatment in both animal models and human patients, has been evaluated [52]. Pathogens in vivo and in situ present lower variation of gene expression, as well as up-regulated genes regarding SOS stress response, alginate biosynthesis and efflux pumps, among others [55]. In that regard, in vitro transcriptional profiles might not have a direct relationship to in situ and in vivo profiles; thus, this technique could open the door to new research that might eventually yield better therapeutics more suited to the in vivo biological phenomena.

## 5. Proteomics

The downstream products of transcripts offer an alternative view on the dynamics of the effect of antibiotics. In particular, there are post-transcriptional mechanisms whose role is often central to understanding which genes actually yield proteins. Moreover, proteins are often the primary targets of antibiotics, so the application of proteomics for MOA identification is well-justified, and as a technique, proteomics is increasingly more prominent in drug discovery [56].

### 5.1. Gel-Based Assays

Early proteomics studies began with gel-based methods, e.g., 2D gel electrophoresis, which profiled the protein constituents of a sample. Comparison of these profiles with the use of fluorophores allowed multiplexing, and as discussed so far, this revealed differential expression [57]. To identify proteins within a certain spot on the gel, digestion followed by mass spectrometry allowed database querying. In line with this, Wang et al. [58] explored the MOA of juglone, a plant-derived 1,4-naphthoquinone, against *S. aureus*. Of the 21 differentially expressed spots, 13 were identified by matrix-assisted laser desorption ionization-time of flight/time of flight (MALDI-TOF/TOF) mass spectrometry. These included proteins that participate in the tricarboxylic acid cycle, DNA and RNA synthesis, and protein synthesis. However, other studies into the MOA of juglone against other microorganisms have revealed considerably different proteomic responses, and juglones’ biomolecular target remains elusive.

Bandow et al. [59] compiled a proteomic profile database of *B. subtilis* response to 30 antibiotics, most of which have been well-characterized regarding their MOA. While each antibiotic presented a complex proteomic profile, with some overlap across similar compounds, a sufficiently unique profile was obtained for each antibiotic. For example, most disrupters of translation accuracy resulted in up-regulation of heat shock proteins, which are induced by the accumulation of misfolded proteins. In the end, 122 proteins with at least a two-fold change in regard to the control samples provided sufficiently distinct profiles to identify the MOA of BAY 50-2369, a novel compound that acts at the peptidyltransferase step of protein synthesis. The identification of the MOA of BAY 50-2369 as a translation inhibitor using proteomics was straightforward in the sense that its proteomic response was similar to other known antibiotics. However, in the case of acyldepsipeptides, their novel MOA could not be extrapolated. Nonetheless, its proteomic profile identified the up-regulation of ClpP and ClpC, two protease subunits, along with down-regulation of the GroEL chaperon and the TU elongation factor, which staged an initial MOA hypothesis. From here, biochemical and chemical genetics assays identified ClpP as the target, which was confirmed with crystallography. Interestingly, acyldepsipeptides do not inhibit ClpP, but release it from ATPase regulation so intact proteins enter its proteolytic chamber, resulting in indiscriminate degradation and cell death [60].

Similarly, Wenzel et al. [61] explored the proteomic response of *B. subtilis* to lantibiotics. More specifically, mersacidin, gallidermin, and nisin were investigated given these bind to lipid II, thereby inhibiting cell wall synthesis. However, mersacidin does not integrate with the cytoplasmatic membrane, while nisin fully integrates and induces the formation of large pores, which impairs membrane potential and leads to nutrient and ion leakage. In between these two ends of the spectrum, gallidermin integrates the membrane, but only induces pore formation in some bacteria. These MOA correlated with proteomic profiles, and when compared with the profiles of other antibiotics that target the bacterial envelope compiled by Bandow et al. [59], YtrE, PspA, and NadE, along with YceC, were revealed as marker proteins of cell wall biosynthesis inhibition, membrane stress, and general cell envelope stress, respectively. Since these marker proteins correlate to the specific steps inhibited, they are indicative of a narrow range of possible molecular targets, and thus enlighten the MOA. More recently, Maaß et al. [62] used proteomic profiling of *Clostridium difficile* to characterize the MOA of metronidazole, vancomycin, and fidaxomicin. Here, 425 protein markers constructed profiles specific to the individual response to each antibiotic, with very little overlap across different antibiotics. Even so, metronidazole affected proteins involved in protein biosynthesis and degradation, DNA replication, recombination, and repair; fidaxomicin altered the expression of proteins with cell envelope functions, cell motility, transcription, and amino acid synthesis; while vancomycin affected a greater diversity of pathways. Although these antibiotics act on different pathways, which validates proteomics as a high-value tool for MOA identification, a higher number of antibiotics should be studied to consolidate the uniqueness of these proteomic profiles. Moreover, testing antibiotics with very similar MOA would allow to test the sensitivity of these profiles in regard to the biomolecular target.

### 5.2. Gel-Free Methods

Gel-based methods are limited regarding throughput and require visual comparison prior to peptide mass fingerprinting. This is a severe limitation when constructing large libraries of proteomic profiles of MOA [63]. Alternatively, one of the preferred gel-free proteomics assays, isobaric tags for relative and absolute quantification (iTRAQ), relies on isobaric peptide labelling for chromatographic separation and their quantification using mass spectrometry [64]. Recent developments in the synthesis and application of isobaric reagents have been revised elsewhere [65]. Other gel-free techniques include stable isotope labelling or selected reaction monitoring [66]. Moreover, enrichment techniques such as antibodies, ionic interaction, or specific enzymes allow the evaluation of post-translational modifications relevant in the infectious process [67]. One of the advantages of iTRAQ is its multiplexing capabilities, whereby commercially available reagents allows testing of 2–8 samples in a single liquid chromatography separation and mass spectrometry analysis [68]. In that sense, Ma et al. [69] quantified the proteomic expression of *S.* *aureus* to daptomycin. In total, 34 proteins were found to be up-regulated, while 17 proteins were down-regulated, of the 872 differentially expressed proteins. Ultimately, the MOA of daptomycin is different from other known antibiotics, and although its exact target remains elusive, two proteins associated with the metabolism of nucleotide acid were validated as a universal response to daptomycin across *S. aureus* strains. Moreover, evidence is building towards cell membrane disruption with sufficient damage to impair its integrity, along with chromosomal aggregation, which results in DNA release and cell death. Although gel-free proteomics has not been extensively applied in the context of MOA characterization of large antibiotic libraries, it has elucidated protein function and cellular interactions. Larger-scale studies, with comparable procedures, are still required before the felt impact of proteomics matches that of previously discussed techniques. In addition to MOA characterization, proteomics has been used to investigate many different determinants of resistance and infection [70], as well as the formation and dynamics of biofilms [71]. Here, the biological processes are evaluated in vivo, as discussed for transcriptomics. One example of such is the iTRAQ study on the cell membrane protein expression of *P. aeruginosa* in cystic fibrosis isolates, which have revealed the host-specific microevolution of this pathogen [72].

## 6. Metabolomics

The last stage of the ‘omics’ cascade focusses on the metabolites present in a biological system. Metabolites are small molecules that belong to different biomolecular classes, including organic acids, amino acids, fatty acids, sugars, sugar alcohols, steroids, and nucleic acids, among others [57]. As such, metabolomics provides a closer representation of phenotype, thus has enormous potential to enlighten the complete dynamics of bacterial physiology in response to antibiotic exposure [73]. Although the metabolome complements the transcriptome and proteome, it reflects cellular activities that are regulated by a wider range of mechanisms. In other words, the metabolome reflects physiological states in a amplified state in comparison with transcriptomics and proteomics [74]. There are two dominant techniques in metabolomics: Nuclear Magnetic Resonance (NMR) spectroscopy and Mass Spectrometry (MS) coupled with a chromatographic step. While at first both had complementary outputs, MS-based techniques have recently become the preferred approach given their higher sensitivity. Even though the last couple of years have seen an increase in NMR spectroscopy-based metabolomics databases, both in quantity and quality, when uncatalogued or unknown metabolites need identification, MS is still the go-to technique [75]. Nonetheless, NMR spectroscopy is able to quantify abundant metabolites, and does not require laborious sample preparation, fractioning procedures, nor derivatization, and can analyze difficult-to-ionize compounds. Moreover, stable isotope labeled NMR spectroscopy can probe the dynamics of metabolite transformation in vivo, as the technique is non-destructive [76].

Regardless of the technique, metabolomics can be targeted or untargeted, the difference being that targeted metabolomics aims to quantify a predetermined subset of metabolites, most often highlighted in previous untargeted analysis. In theory, untargeted metabolomics is more suited for MOA identification, mostly because no a priori knowledge is required. In practice, a targeted metabolomics protocol can equally predict MOA, as such this distinction was not discussed in the following study reviews.

### 6.1. Nuclear Magnetic Resonance Spectroscopy

Kozlowska et al. [77] employed 1H high-resolution magic angle spinning NMR spectroscopy to identify the lowest concentration of antimicrobial peptides that induced a detectable metabolic response in *E. coli*. Although this study did not aim to discriminate different MOA, its rational was to detect antibiotic-specific responses but also avoid large scale death cell and its associated non-specific response. The authors tested four structurally similar, and physically related, cationic amphipathic antimicrobial peptides with different degrees of activity. Unique metabolic responses for each compound at sub-inhibitory concentrations were found, and these were not coherent with observed MIC and recovery assays, which suggest some sort of phenomena related to the ratio between antibiotic and bacteria, and goes to show the gap between observable phenotype and underlying biological mechanisms.

Hoerr et al. [78] used NMR spectroscopy to study both the intracellular fingerprint and extracellular footprint of *E. coli* when exposed to nine antibiotics from five classes, which inhibited protein synthesis, nucleic acid synthesis, or cell wall biosynthesis. While antibiotics acting on intracellular targets consistently presented fingerprints coherent with class-action, only antibiotics acting on the cell wall had a distinct metabolic footprint (i.e., specific molecular signatures of the extracellular media), which has been associated with the loss of membrane integrity and subsequent metabolite leakage. As such, metabolic fingerprinting is a more relevant approach to explore antibiotic MOA, despite the fact that it is more laborious. More importantly, a descriptive model built from the metabolic profiling data was used to successfully predict the MOA of antibiotics not used in the training dataset. Even so, further studies with more MOA diversity, as well as larger libraries, were deemed necessary.

Interestingly, Birkenstock et al. [7] conducted a exometabolome analysis using 1H NMR spectroscopy, which determined the target of triphenylbismuth dichloride as pyruvate dehydrogenase in *S. aureus*. This analysis revealed that pyruvate concentrations strongly increase with exposure to triphenylbismuth dichloride, along with detectable suppressive effects on glucose and amino acid consumption, as well as reduced accumulation of acetate. Additionally, acetolactate, acetoine, butanediole, lactate, formate, and ethanol also accumulated, which indicated that accumulated pyruvate was directed to alternative pathways. As such, this antibiotic was suspected of interfering with pyruvate catabolism, which was the basis for further enzymatic analysis to pinpoint the exact target.

### 6.2. Mass Spectrometry-Based Methods

Regarding MS-based metabolomics, two separation techniques are typically employed prior to mass spectra acquisition, Gas Chromatography (GC) and Liquid Chromatography (LC). Small molecules in general, and metabolites in particular, are subject to high temperatures in the preparation and analysis with GC-MS. In the case of blood plasma, this alters the molecular peak pattern by up to 40%, including the formation of degradation and transformation products [79]. Because of this, and given LC achieves better separation, the focus of this section was placed on LC-MS.

Schelli et al. [80] compared the metabolic response of a methicillin-resistant and susceptible strain of *S. aureus* to sub-inhibitory concentrations of ampicillin, kanamycin and norfloxacin, which belong to the classes of β-lactams, aminoglycosides, and quinolones, respectively. Interestingly, minor differences were induced in the sensitive strain by kanamycin and norfloxacin, but marked differences were induced by ampicillin. A similar pattern was observed on the resistant strain; however, these differences in metabolic profile were more prominent across the three antibiotics with the susceptible strain. In total, 109 and 107 metabolites were significantly altered by antibiotic exposure for the sensitive and resistant strains, respectively. Moreover, Principal Component Analysis (PCA) score plots of the metabolic response showed moderately good separation according to class. On the downside, dispersion of untreated samples with either strain highlighted a critical limitation of MS-based metabolomics, namely its high sensitivity often masks the MOA-related signal.

Zampieri et al. [17] probed the initial response (1 min to 1 h) of *E. coli* to 9 antibiotics and hydrogen peroxide. Here, 324 putatively annotated metabolites were significant altered in comparison with untreated samples, out of 784 detected. In general, antibiotic exposure had an extensive effect on metabolism. Not only did antibiotic exposure alter a large number of metabolites, but 37 of these displayed similar patterns after exposure to at least two compounds with considerably different MOA. Even so, because not all significantly altered metabolites displayed the same pattern across all antibiotics, as well as the fact that all samples exposed to antibiotic with similar MOA had at least one exclusive responsive metabolite, except for amoxicillin and ampicillin, the authors highlight the suitability of MS-based metabolomics to unravel the MOA of antibiotics.

One limitation of metabolomics techniques is its high sensitivity to subtle between-sample variations. For instance, some antibiotics are better dissolved in particular solvents, and this often masks the MOA-related signal that is the objective of these studies [78]. Another limitation regards peak annotation using databases of known metabolites [81]. In addition, metabolomics is still limited regarding throughput, although a 10–100× gain can be achieved regarding proteomics, which requires peptide fragmentation and more demanding acquisition conditions [17]. Although chromatography-free MS-based metabolomics can achieve a throughput of 10,000 samples/day with a single flow injection electrospray equipment, cycle time was expected to be reduced to anywhere between 4–8 s in the near future [82].

In that regard, Zampieri et al. [83] optimized a chromatography-free protocol for rapid metabolome profiling, relying on microtiter plates, thus suitable for automation and increased throughput. Using direct flow injection high-resolution MS, MOA profiles of 62 reference compounds were gathered with *Mycobacterium smegmatis*. These served to extrapolate the MOA of 212 compounds of a GSK antibacterial library, a few of which were experimentally validated on *M. tuberculosis*. Despite the scale of this study, only 8% of said library targeted unconventional cellular processes, e.g., those involving the trehalose and lipid metabolism, an indication of the challenge that is to discover novel MOA. Importantly, MS-metabolomics identified a large range of MOA, some of which were putative, even in the absence of growth inhibition. However, discerning drug-target effects from indirect metabolic adaptations is difficult and requires further investigation, for instance, with transcriptomics, chemical genetics, or biochemical assays.

## 7. Bacterial Cytological Profiling

The evolution of technologies such as electron and fluorescence microscopy has been critical towards understanding various microbial processes, including those between pathogens and their host. Importantly, the simultaneous acquisition of multiple parameters from microscopy images enabled profiling the MOA of antibiotics towards their prediction [84]. First introduced by Giuliano et al. [85], High-Content Screening (HCS) was proposed as a technique for drug discovery that combined high-throughput screening with a tabletop instrument capable of reading up to four channels of fluorescence at sub-cellular resolution. HCS was originally demonstrated with the drug-induced transport of GFP from the cytoplasm to the nucleus of human tumor cells. Several hurdles had to be cleared for HCS to be applied to prokaryotes. For instance, bacterial cells lack the organelles for which fluorescent labels were developed, and the high-throughput image-based screening technologies were not suitable at the magnifications required to analyze bacterial cells. In addition to this, because of their smaller size, fluorescent signals were generally weaker, which complicated subsequent analysis. Nonetheless, the rationale was that antibiotic-induced morphological changes would be indicative of the MOA of antibiotics [6].

One of the first attempts at HCS of bacteria was described by Peach et al. [86], who used epifluorescence microscopy to build cytological profiles of intertwined monolayers of *Vibrio cholerae* challenged with a set of 58 FDA-approved antibiotics with known MOA. Using feature segmentation and extraction, key structural metrics composed cytological profiles, from which the MOA of a natural product library was estimated. These profiles identified the three major pathways affected by antibiotics, namely protein, DNA, and cell wall synthesis. Although cytological profiles were unsuitable to pinpoint the exact molecular target, these were beneficial in large-scale screening campaigns as a triage step applicable at early stages of discovery programs, thereby minimizing and guiding subsequent testing [87]. Similarly, and nearly simultaneously, Nonejuie et al. [13] coined this technique as Bacterial Cytological Profiling (BCP), and used it to ascertain the MOA of 41 antibiotics, of 26 structural classes, on *E. coli*. Firstly, the profiles induced by inhibitors of transcription, translation, DNA replication, lipid, and peptidoglycan synthesis were gathered, and these presented considerable differences that served to distinguish among them. Then, cytological profiles induced by antibiotics of different classes, but inhibiting the same pathway, were clustered. Namely, three clusters of antibiotics were found for protein synthesis inhibitors: those that completely block elongation, those that promote mistranslation and alter membrane permeability, and those which result in premature termination. Therefore, cytological profiling seemed suitable to discriminate the effect of compounds beyond protein synthesis inhibition. A similar result was found for the remaining major pathways targeted, albeit MOA could not be resolved in finer detail.

Although there had been previous studies applying fluorescence microscopy to reveal the biological effects of antibiotics [88], the notion of comparing cytological profiles to provide insight into the MOA of antibiotics was first reported by Lamsa et al. [89]. However, it was the work of Peach et al. and Nonejuie et al. who consolidated BCP as a primary screening technique for antibiotic discovery from large libraries, and for tangential objectives likewise, such as for susceptibility testing [90]. Most importantly, BCP has been adopted by the ‘big pharma’. For instance, McLoed et al. [91] used BCP to further validate the suspected MOA of a promising compound identified after a high-throughput phenotypic screen of ~1.2 million compounds of the AstraZeneca compound collection against *E. coli*. An alternative use of BCP was described by Zoffman et al. [12], who aimed to identify the lowest concentration of antibiotics that induced significant changes to bacterial phenotype. Because phenotypic changes were detected under the MIC, this approach effectively expanded the ‘screenable’ space of the compound library to include the grey chemical matter, which is characterized by limited activity that could be improved with medicinal chemistry efforts, and eventually reach the clinic.

## 8. Vibrational Spectroscopy

Vibrational spectroscopy (VS) is based on chemical bonds having a unique vibrational energy. Thus, a samples spectrum reflects the vibrational modes of major cellular biomolecules, such as proteins, carbohydrates, lipids, or nucleic acids. This provides a sensitive and complete metabolic fingerprint, which can be obtained using simple, rapid, reagent-less, and label-free procedures [92]. Within VS, two techniques hold great potential: Raman Scattering (RS) and Fourier-Transform Infrared (FTIR) spectroscopy (FTIRS). FTIRS measures the vibrational modes of molecular bonds that result from dipole moment changes, i.e., charge differences in the electric field of atoms. RS probes electric polarizability changes, so it is often complementary to FTIRS. While these are far from generating comprehensive metabolite-level data on the metabolome, the information provided is sufficiently revealing of the metabolic networks involved to be applied for metabolic fingerprinting [74], and ultimately MOA-centric studies.

### 8.1. Raman Scattering

López-Díez et al. [93] investigated the effect of amikacin, an aminoglycoside antibiotic, on *P. aeruginosa* using UV resonance RS spectroscopy, which is particularly suitable to probe nucleic acids and aromatic amino acids. Qualitative and quantitative multivariate analysis on the concentration-dependent effect of amikacin revealed that, as the concentration increased, there was a shift from protein-associated bands towards nucleic acid peaks. This finding is coherent with the MOA of amikacin, which binds to ribosomal RNA, resulting in the misincorporation of amino acids and therefore inhibition of translation. As protein synthesis is repressed, there is an accumulation of nucleic acids and reduction of proteins in the cell. Similarly, Athamneh et al. [94] employed RS spectroscopy to evaluate the antibiotic response of *E. coli* to 15 antibiotics of 5 classes. Firstly, the objective was to discriminate each antibiotic’s effect using a PCA followed by a linear discriminant analysis, which achieved an accuracy of 83.6%. Then, some antibiotics were held out from the model building and the analysis was repeated, yielding a less impressive accuracy of 48%. While this study is a more robust proof-of-concept than the previous, given a more comprehensive set of antibiotics and their MOA were considered, the question whether a truly novel MOA could be predicted remains. On a slightly different note, Liu et al. [95] explored surface-enhanced RS spectroscopy, which is particularly adequate to study low-abundance molecules, towards the susceptibility testing of both *E. coli* and *S. aureus*. While this study was not aimed at ascertaining the MOA, several RS spectra alterations were coherent with the MOA of antibiotics. Although this issue was not fully considered, it suggested the possibility of MOA identification with this technique. A similar conclusion was reached by Teng et al. [96], who explored single-cell RS to differentiate the *E. coli* stress response to ethanol, ampicillin, kanamycin, n-butanol, and heavy metals. Here, RS bands were related with distinct stress responses, and more importantly with the mechanism of the stressor. Using a combination of RS and transcriptomics, Germond et al. [97] predicted the acquired resistance mechanisms of 10 laboratory-evolved strains *E. coli*, even in the absence of antibiotics. Interestingly, a linear relationship was found between resistance mechanism-associated bands and the expression levels of the genes known to grant resistance to the action of antibiotics.

### 8.2. Fourier-Transform Infrared Spectroscopy

Nguyen et al. [98] explored FTIRS in combination with RS to individually compare *E. coli* control samples with those exposed to ampicillin, cefotaxime, tetracycline, and ciprofloxacin. Interestingly, both FTIRS and RS distinguished the control samples at different time-points (3, 6, 8, and 24 h), with RS revealing more bands indicative of growth phase. Regarding the antibiotic-exposed samples, altered bands detected with FTIRS include those associated with carbohydrates and proteins, while those detected with RS were associated with nucleic acids and phenylalanine. Unfortunately, a simultaneous comparison of all antibiotics was not presented, so evaluating the ability of either FTIRS or RS to ascertain MOA is challenging. Even so, it is interesting to note that the standard deviation of technical replicates was higher with RS than with FTIRS. On a slightly different note, Huleihel et al. [99] used FTIRS to differentiate the effect of caffeic acid phenethyl ester (CAPE), a natural honeybee product with potent antimicrobial activity, and ampicillin, on 9 Gram negative and 8 Gram positive bacteria. The effect of CAPE was remarkably different on Gram negative and Gram positive bacteria, which could be due to its biological effect. An increase of the bands associated with proteins and sugars, along with a major reduction of the band associated with nucleic acids, was observed in Gram positive bacteria. Similarly, an increase of the band associated with sugar content, along a decrease of the protein-associated band, was detected in Gram negative microbes.

FTIRS has been underexplored for MOA classification, but a few examples with tangential objectives can be brought to the discussion. Moen et al. [100] compared the global transcriptomic profile and FTIR spectra of *E. coli* when challenged with 10 adverse conditions. Although 40% of the 4279 genes investigated had differential expression, no correlation between the transcriptional profile and the biomolecular profile obtained with FTIRS was found. Nonetheless, a PCA on the spectral regions associated with fatty acids, proteins, and carbohydrates revealed stress-induced sample separation. Similarly, Corte et al. [101] developed a FTIRS toxicity assay with *Saccharomyces cerevisiae*, for which various spectral stress indexes were developed. While their study had an ecotoxicology focus, the stress response to ethanol, sodium hypochlorite, sodium chloride, and sulphur dioxide at low concentrations and after a short exposure was clearly captured on the FTIR spectra. Because FTIRS has been underexplored but holds great potential, we dwelled into its application towards MOA identification. Firstly, we applied a macro-cultivation assay, from which it became clear that metabolic fingerprints reflect the MOA of antibiotics [102]. We then refined our assay into a high-throughput micro-cultivation protocol, from which we successfully predicted the MOA of antibiotics at the level of the major biosynthetic pathway, class, and individual antibiotics. Moreover, MOA was accurately predicted, at all levels, when models were trained with similar samples, to simulate cases of rediscovery, and when models were trained without similar samples, to simulate novelty. Our assay seems to be suitable to probe the grey chemical matter, as when the dose-response of MOA prediction was determined, an average growth inhibition of 15% yielded 70% accuracy at MOA prediction. Lastly, using spectra that were obtained from normalized samples regarding biomass, we were able to predict growth inhibition, which suggested that metabolic fingerprints obtained with FTIR spectra have intrinsic patterns that reflect growth inhibition beyond cell density, and opened the door to a single-step assay that simultaneously predicts MOA and potency [103].

## 9. Conclusions

**Figure 3:**
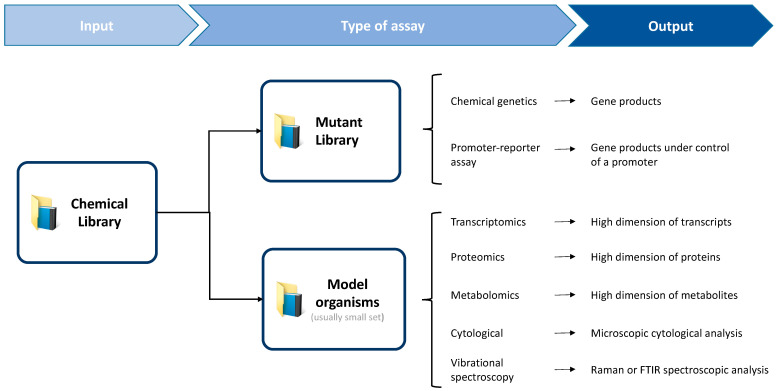
Two commonly employed approaches to predict antibiotic MOA. These are typically based on the high-throughput screening of a chemical library against a high dimension mutant bacteria library, or against a smaller set of model bacteria. Accordingly, different types of assays are applied, which generate specific outputs that are often complementary.

Throughout this review, the application of various techniques to MOA identification and characterization have been described (Figure 3). Given MOA identification is a major bottleneck of the phenotypic screening approach, and that phenotypic screening has been the most successful in delivering first-in-class antibiotics, techniques for MOA identification are increasingly important. The focus of this review was on techniques capable of both sufficient throughput to be employed in screening campaigns of large libraries, and sufficient sensitivity to accurately distinguish antibiotics MOA. Additionally, given an increasing appreciation that antibiotics elicit system-wide responses, emphasis was put on techniques that output a holistic MOA profile.

While mutants of overexpression or knockdown/knockout libraries evaluate a gene at a time, a single reporter strain probes the genes under the regulation of a promoter (Figure 2). Promoter-reporter systems share some advantages with mutant libraries, but also some disadvantages. While the readout is also simple and can be associated with a cluster of genes, collections comprise a large number of reporter strains (e.g., >1500), crippling the throughput of this technique. Some strategies attempted to reduce the number of strains to a minimum while obtaining relevant profiles. However, additional disadvantages include the background noise of fluorescent labels, or an inability to probe post-transcriptional events, which results in a biological gap between gene transcription and phenotype that this technique does not cover.

An alternative to reporters of gene transcription is to probe the transcriptome. The advantage is that transcriptional profiles provide a system-level readout, but this made the biological interpretation of the results more challenging. Regarding hybridization-based transcriptomics, these are generally lower-cost and their multiplex capabilities imply a good degree of throughput. However, only the genes used to build the microarray can be detected, and issues arise due to cross-hybridization, as well as with low-abundance transcripts. Beyond hybridization techniques, those based on sequencing have seen their cost steadily decrease into reasonable levels, which enables a more commonplace usage. In addition to detecting all transcripts of a sample, RNA-seq allows querying post-transcriptional events, which is a step forward towards closing the gap between gene transcription and phenotype.

Most antibiotics target proteins. As such, proteomics is well-suited to investigate MOA. Additionally, because proteins are the end-product of genes, the effect of post-transcriptional and post-translational regulatory mechanisms can be queried, which is a step closer to understanding the biological mechanisms ruling antibiotic-induced phenotype. While proteomics does not require manipulated strains, it is generally a laborious, low-throughput, and challenging technique. Despite their critical role in understanding bacterial physiology, and the MOA of antibiotics, proteomic profiles are less distinctive of MOA than transcriptomics. In addition, the throughput of proteomics has not reached that of metabolomics. Consequently, there have been fewer studies applying proteomics to illuminate the MOA of large antibiotic libraries.

The last component of the Omics cascade are metabolites, which have higher variability in terms of structure and biological function, but are a closer reflection of phenotype than proteins, transcripts, and genes. As such, metabolomics can not only pinpoint an antibiotics’ target, but also identify the MOA of antibiotics that do not target the metabolism. Despite a few promising protocols having 10–100× the throughput of proteomics, high-throughput metabolomics-based MOA identification is still in its infancy. For NMR spectroscopy, low metabolome coverage and fewer annotation resources are common issues. Regarding MS-based methods, the optimization of chromatographic separation, peak annotation, and masking of MOA-related signal by subtle undesirable sample variations are still major bottlenecks. Nonetheless, MS-based methods have been successfully applied to large compound libraries, which validates its use for screening of large antibiotic libraries.

An alternative to the techniques discussed thus far lies in BCP, which has been developed for eukaryotic cells. Although its application to prokaryotes only recently gained traction, the ultrastructural and morphological alterations induced by antibiotics yield a cytological profile with good predictive ability of the MOA of antibiotics. The issue with this approach, which has a different perspective on the issue of MOA, is one of sensitivity. While the aptitude to detect the major pathway affected has been well established, contradictory findings have been reported regarding the ability to separate profiles induced by drugs that act on the same pathway, and it is still unclear if BCP can be used as a standalone technique or requires complementation with biomolecular techniques. Nonetheless, its high- to very high-throughput has made HCS an attractive technique, rightfully justifying further efforts towards its consolidation in the discovery pipeline.

Another alternative is VS, which has been increasingly gaining traction for metabolic fingerprinting. Similarly to BCP, this technique is promising because it enables adequate throughput for screening purposes with sufficient biological information for MOA identification. However, because it reflects the biochemical composition of the sample, it has the potential to be more informative than BCP, without requiring complementary assays. Furthermore, because these techniques are high-throughput, reagent-less, label-free, and involve reduced or no sample preparation, they hold tremendous potential. Even so, few studies have explored large antibiotic libraries, which by itself justifies a deeper evaluation on the application of vs. towards antibiotic discovery.

Due to the lack of novelty in the antibiotic pipeline, and the desperate need for new antibiotics, it has become increasingly evident that a new approach is needed. The ability of the various Omics techniques discussed, as well as BCP and VS, to rapidly and robustly detect MOA could enable a shift from the activity-based antibiotic discovery paradigm towards a mechanism-based approach. An advantage of a mechanism-based approach is that, in the absence of extensive growth inhibition, the pool of positive hits is reduced to those that are more directly related to the antibiotic-target interaction. This is because when probing the cell’s response to antibiotics, for example, with differential mRNA or protein expression, different transcriptional or translational levels strongly reflect a state of reduced cell metabolism, energy production, DNA, RNA, or protein synthesis levels, which are first and foremost a consequence of reduced bacterial growth. Identifying the specific target from said hit pool is often treacherous, if not impossible. Because of its bias towards high potency compounds, the activity-based approach is a suboptimal approach to MOA identification with arguably unsurmountable limitations. In addition, a mechanism-based discovery approach increases the ‘screenable’ space by probing the grey chemical matter, an underexplored source that is extremely promising because medicinal chemistry can improve the properties of these compounds, in particular their potency. Here, a low-cost high-throughput technique for MOA identification can better guide this process by rapidly excluding off-target liabilities. With a higher number of higher quality hits, and their better guided structural optimization, it is expected that new compounds can eventually reach the clinic and spark a new generation of antibiotics.
